# Energy Partitioning in Multicomponent Nanoscintillators for Enhanced Localized Radiotherapy

**DOI:** 10.1021/acsami.3c00853

**Published:** 2023-05-12

**Authors:** Valeria Secchi, Francesca Cova, Irene Villa, Vladimir Babin, Martin Nikl, Marcello Campione, Angelo Monguzzi

**Affiliations:** † Dipartimento di Scienza Dei Materiali, Università Degli Studi Milano-Bicocca, 20125 Milano, Italy; ‡ NANOMIB, Center for Biomedical Nanomedicine, University of Milano-Bicocca, P.zza Ateneo Nuovo 1, 20126 Milan, Italy; § FZU—Institute of Physics of the Czech Academy of Sciences, Cukrovarnická 10/112, 16 200 Prague, Czech Republic; ∥ Department of Earth and Environmental Sciences, Università Degli Studi Milano-Bicocca, Piazza Della Scienza 4, 20126 Milano, Italy

**Keywords:** radiotherapy, scintillators, energy transfer, singlet oxygen, nanomaterials

## Abstract

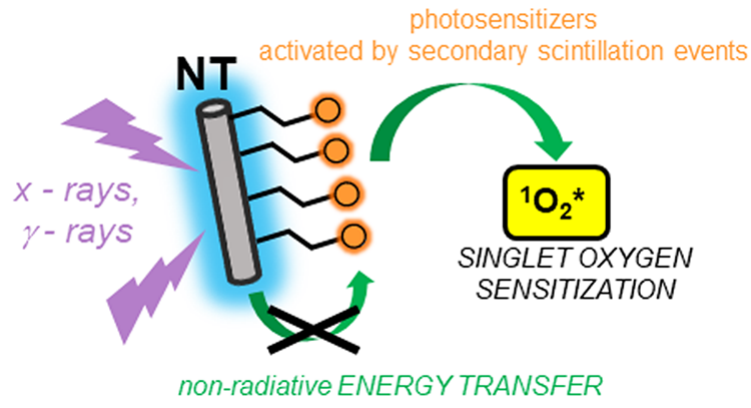

Multicomponent nanomaterials consisting of dense scintillating particles functionalized by or embedding optically active conjugated photosensitizers (PSs) for cytotoxic reactive oxygen species (ROS) have been proposed in the last decade as coadjuvant agents for radiotherapy of cancer. They have been designed to make scintillation-activated sensitizers for ROS production in an aqueous environment under exposure to ionizing radiations. However, a detailed understanding of the global energy partitioning process occurring during the scintillation is still missing, in particular regarding the role of the non-radiative energy transfer between the nanoscintillator and the conjugated moieties which is usually considered crucial for the activation of PSs and therefore pivotal to enhance the therapeutic effect. We investigate this mechanism in a series of PS-functionalized scintillating nanotubes where the non-radiative energy transfer yield has been tuned by control of the intermolecular distance between the nanotube and the conjugated system. The obtained results indicate that non-radiative energy transfer has a negligible effect on the ROS sensitization efficiency, thus opening the way to the development of different architectures for breakthrough radiotherapy coadjutants to be tested in clinics.

## 1 Introduction

Looking at recent research, it is clear that nanotechnology can play an important role in the biomedical science thanks to the successful development and use of nanoparticles for theranostics, diagnostics, monitoring of specific injured tissues or organs, and for the improvement in some traditional therapeutic treatments.^1−3^ This is mainly due to the advantages of nanomaterials with respect to bulk systems, such as the facile surface functionalization, the composition versatility, and their tailorable optical and magnetic properties, which allow them to respond to the specific demands of the targeted application and use. Consequently, a huge family of nanomaterials, such as metallic and semiconductor nanoparticles, metal/lanthanide oxides, and organic and hybrid systems, has been developed to be used in advanced diagnostic and imaging techniques, drug delivery strategies, or innovative therapeutic approaches against cancer and other deadly diseases,^4−7^ as demonstrated by the increasing number of nanosystems approved by the Food and Drug Administration agency.

For example, we can observe an increasingly larger use of radioluminescent nanoparticles, i.e., nanoscintillators, able to absorb and convert the ionizing radiation (*X*- or γ-rays) into a large number of UV–visible photons, which are exploitable to boost the efficacy of diagnostic techniques for preclinical mapping, intraoperative imaging, radiation dosimetry, and, importantly, as efficient coadjutants in oncological therapies.^8−11^ The search for innovative therapies to surpass state-of-the-art treatments is indeed still highly persistent. The standard cancer treatment options, represented by chemotherapy, radiotherapy, and surgery, are still associated with systemic side effects, disease recurrence, and drug/radio resistance of malignant cells. Among them, the radiotherapy exploits the effect of the ionizing radiation that directly damages the cellular DNA or indirectly forms cytotoxic reactive oxygen species (ROS), such as hydroxyl radicals and singlet oxygen (SO), upon interaction with the intracellular aqueous environment.^7,12^ However, radiotherapy is strongly limited by the maximum radiation dose that can be given to a tumor mass without incurring significant injuries to the adjacent tissues or organs.^13^ Modern approaches envisage the use of patient-specific dose-delivery plans or short radiation pulses to limit collateral effects,^14,15^ but these strategies does not solve the problem of absolute lack of selectivity of the ionizing radiation for the sick tissues. In this regard, the photodynamic therapy (PDT) has been proposed as an alternative to radiotherapy due to its high selectivity and minimal invasiveness.^16^ PDT exploits indeed specific photosensitizer (PS) moieties which are selectively activated only by light in the presence of molecular oxygen in order to produce ROS.

The PDT has been utilized in the clinic for treatments of different cancer types, but despite the excellent results obtained, its clinical use is actually hindered by the shallow tissue penetration of light, especially in the visible spectral window where most of the best PSs absorb the electromagnetic radiation.^17,18^

**Figure 1:**
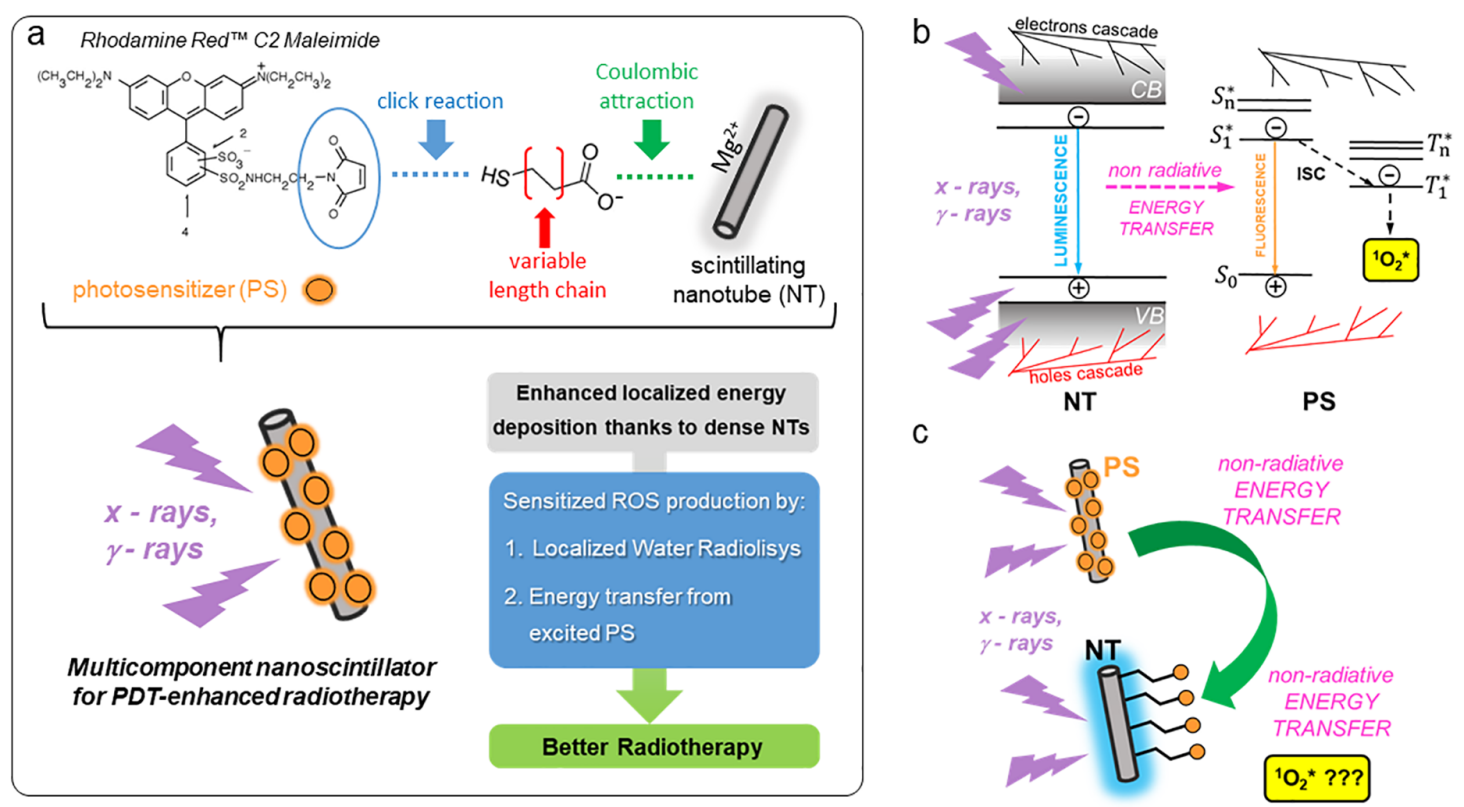
(a) Synthesis of multicomponent scintillating nanotubes (NTs) for PDT-enhanced radiotherapy achieved by the use of an SO () PS. (b) Photophysics of the sensitization of SO production under exposure to ionizing radiation. The free electrons and holes generated by interaction between the ionizing radiation and the NT recombine directly on the NT and on the PS. The latter is promoted to its excited-state singlet (S_n_*) or triplet (T_n_*) with a ratio of 1:3. The energy stored in the NT can be therefore transferred by non-radiative energy transfer (ET_nr_) producing additional PS molecules in the S_1_* state. The PS molecules in the S_1_* state can subsequently experience intersystem crossing (ISC) that further populates the T_1_* state. From PS in the triplet state, the energy is transferred by a second non-radiative energy transfer step to molecular oxygen, which is promoted to its excited singlet state . (c) Sketch of ET_nr_ active and ET_nr_ inactive multicomponent scintillating NTs realized by incrementing the intermolecular distance between the NT and the PS molecules.

An important step forward to overcome both radiotherapy and PDT drawbacks was made in 2006, with the introduction of the concept of energy transducers to transform the energy deposited by X-rays or γ-rays into optical-range luminescence.^19^ The core of this PDT-enhanced radiotherapy is the use of luminescent dense nanoscintillators that can interact efficiently with the ionizing radiation achieving also a photon down-conversion into the visible range to activate the PSs, by both radiative and non-radiative energy transfer processes (Figure 1a).^20−22^ The presence of these nanoscintillators allows (i) the promotion of localized energy deposition in the tissue of interest and (ii) the activation of the PDT effect in deep tissues^23−25^ by means of a complex energy partitioning scheme. To date, diverse classes of inorganic dense nanoscintillators have been combined with organic PSs,^26^ and they have been investigated both in vitro and in vivo.^12,27^ The excellent results obtained demonstrate clearly that this approach results in a synergistic therapeutic effect of radiotherapy and PDT,^28−30^ thanks to the enhanced sensitization of ROS production given by the presence of PS systems.^20^ Nevertheless, a complete understanding of the energy partitioning that occurs in the scintillation process among the dense nanoscintillators, the PDT agent, and the biological environment is still lacking. Consequently, the general guidelines for the design of optimized nanomaterials to be tested in a clinical environment are still eagerly required.

Here, we studied the role of the non-radiative energy transfer (ET_nr_) process between the nanoscintillator and the PDT agents in the global energy partitioning mechanism. Parallel to the passive sensitized activation, triggered by the presence of the dense nanoscintillator that enhances the localized release of the ionizing radiation energy (Figure 1b), ET_nr_ is indeed usually considered a crucial activation pathway for PDT sensitizers,^12,31^ but no direct proof has been given yet.^28^ Considering that the optimization of ET_nr_ imposes several severe restrictions on the material composition, architecture, and electronic properties in order to couple effectively a nanoscintillator to a PS system, it is therefore crucial to understand its effective role in the global sensitization process for the design of optimized radiotherapy coadjutants. In particular, we investigate a series of scintillating nanotubes (NTs) functionalized with a model conjugated PS for the production of singlet oxygen (SO). The ET_nr_ rate and yield have been finely tuned by controlling the intermolecular distance between the NT and the chemically coupled PS molecules. The results obtained suggest that ET_nr_ has a minor role in the SO sensitization process, thus opening the way to the development of different architectures for highly effective radiotherapy coadjutant to be tested in clinics.

## 2 Results and Discussion

**Figure 2:**
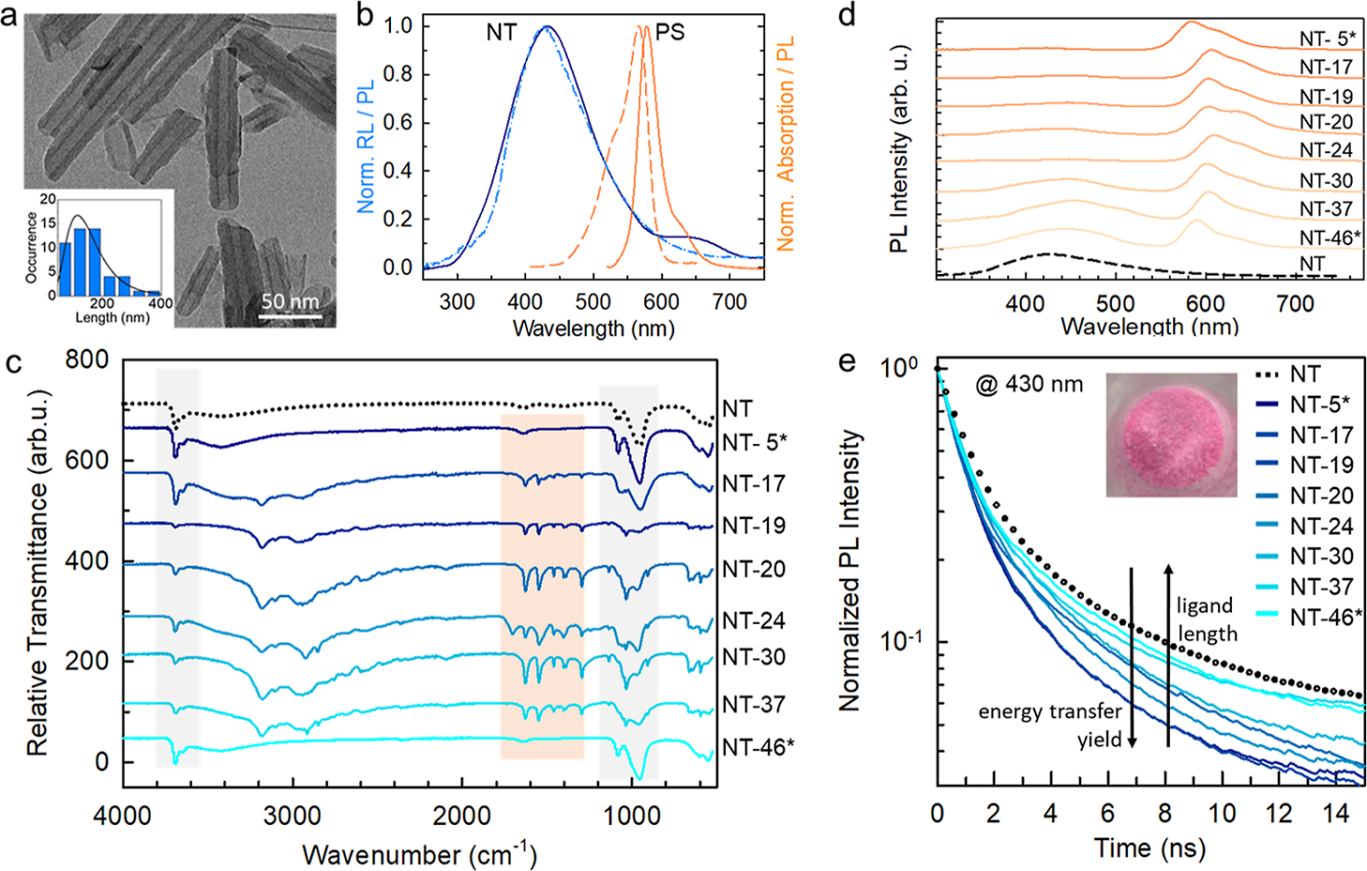
(a) Transmission electron microscopy (TEM) image of scintillating chrysotile NTs and their size distribution (inset). (b) On the left, the PL (dashed–dotted line, exc. 250 nm) and radioluminescence (RL, solid line) spectrum of the NT under soft X-ray exposure (dashed–dotted line). On the right, absorption (dashed line) and PL (solid line) spectra of the conjugated chromophore Rhodamine Red C_2_ maleimide selected as the model SO PS. (c) Attenuated reflectance FT-IR spectra of NTs and the multicomponent nanoscintillator series obtained by tuning the PS-to-NT intermolecular distance from 5 Å (NT-5*) to 46 Å (NT-46*). The asterisks mark the sample where Rhodamine Red C_2_ maleimide is substituted with rhodamine B. Shaded areas mark the characteristic IR mode of the NT (gray at around 1000 and 4700 cm^–1^) and of the PS (orange, 2300–2500 cm^–1^). (d) PL of multicomponent nanoscintillators as a function of the NT-to-PS intermolecular distance under UV excitation at 250 nm. The spectra are normlized to the PS emission peak in the red spectral range. (e) PL intensity decay in time recorded at 430 nm under pulsed excitation at 250 nm of NTs and the functionalized NT sample series. The inset is a digital picture of the NT-20 sample under daylight.

As detailed in the Experimental Methods section, the PS-functionalized nanoscintillators have been realized by coupling biocompatible chrysotile NTs to the conjugated chromophore Rhodamine Red C_2_ maleimide by means of several heterobifunctional bridges of different lengths (Figure 1a, see Supporting Information file, Table S1, Supporting Figure S1). The NTs have been synthesized in aqueous solution under hydrothermal conditions in the presence of Mg and Si precursors. We obtained pure chrysotile NTs (Figure S2) of diameter 50 nm and average length 100 nm (Figure 2a) with a blue scintillation and photoluminescence (PL) peaked at 430 nm (Figure 2b). The external surface of the NTs is brucitic,^32^ showing a positive ζ-potential which allows the coulombic interaction with anionic species such as the carboxyl functional group at one end of the bridge ligand series employed (Figure 1a). The PS system has been selected because of (i) a suitable energetic resonance between its ground state absorption and the NT scintillation emission (Figure 2b), which allows the occurrence of non-radiative ET_nr_ by both the Dexter and Förster mechanisms between the NT and the PS molecules,^33^ and (ii) the presence of the maleimide functionality. The latter is a crucial point because this functionality allows us to exploit the thiol–maleimide click reaction with the −SH functional group at one end of the NT surface ligand to anchor the PS (Figure 1a), therefore controlling their compositon.^34,35^ So, although the resonance with the NT emission is not ideal, the employed PS is the ideal system to perform the designed experiments. In such a way, by varying the length of the connecting ligand, we can tune the rate and yield of ET_nr_ by increasing the intermolecular distance between the NT and PS from 17 to 37 Å (Table S1). The different samples are labeled as NT-*x*, where *x* is the distance between the NT and the PS expressed in angstroms. It is worth noting that the organic ligand employed is not rigid; so, the considered intermolecular distances are nominal values taken as the reference. To have also very short or very large intermolecular distances of 5 and 46 Å, we used as the PS the conjugated chromophore rhodamine B (Figure S1) that possesses the right anionic functionality to be directly anchored on the NT surface (NT-5*)^36,37^ or placed quite far by using polyethylene glycol as the connecting ligand (NT-46*). Considering the typical non-radiative interaction radii and the poor luminescence yield of NTs,^33^ in sample NT-5*, the ET_nr_ yield ϕ_ET_^nr^ should be maximized while minimized in the sample NT-46*. In the latter case, given the limited energetic resonance between NT emission and PS absorption, the contribution of ET_nr_ to the SO sensitization can be for sure neglected and therefore completely decoupled from the other mechanisms involved (Figure 1b).

**Figure 3:**
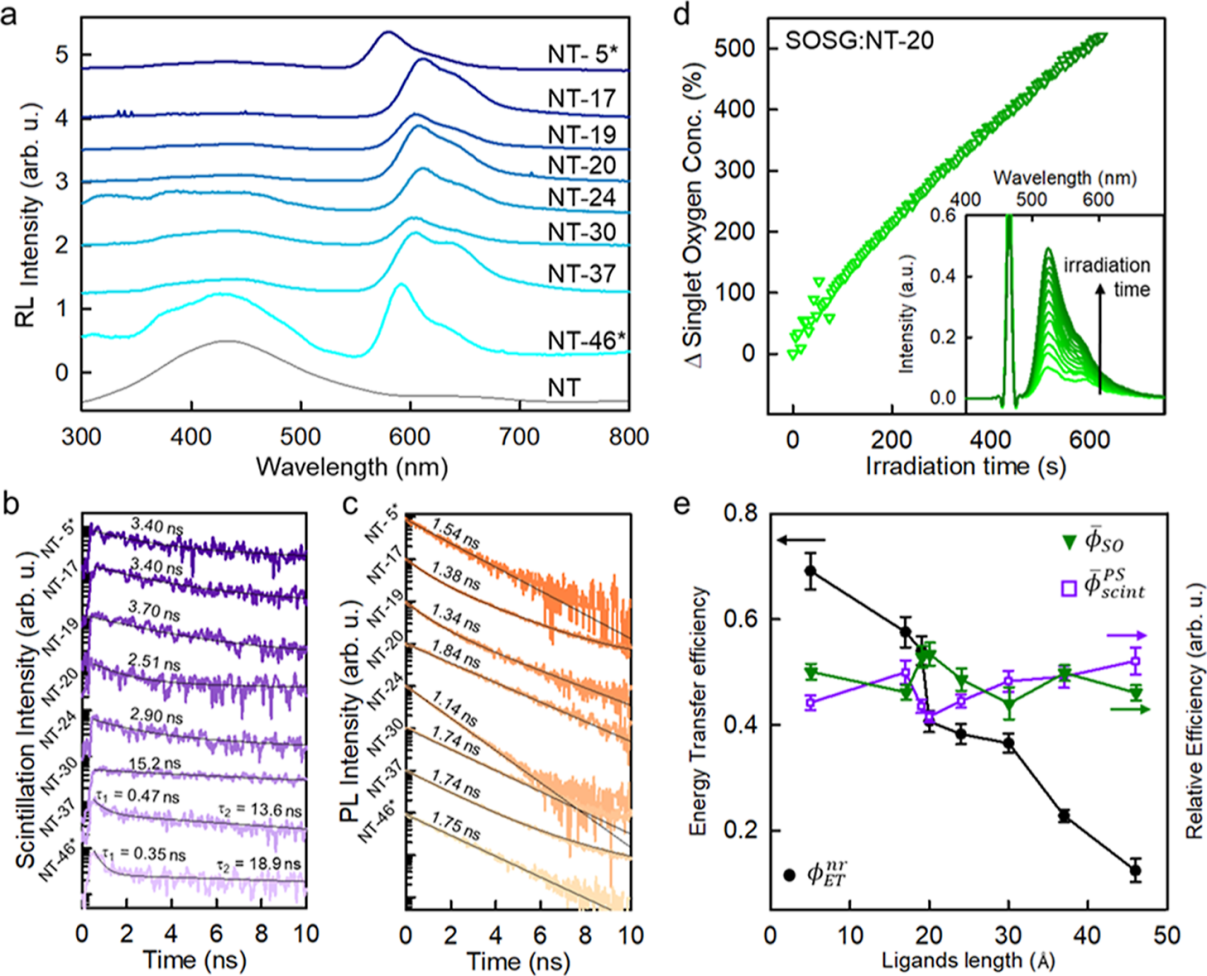
(a) RL spectra of the multicomponent nanoscintillator series as a function of the PS-to-NT intermolecular distance, normalized to the residual PS emission intensity. (b) Scintillation pulses recorded at ca. 620 nm under pulsed X-ray excitation at 40 keV. (c) PL intensity decay at 620 nm under pulsed excitation at 250 nm. (d) Relative increment of the SO concentration as a function of the irradiation time under soft X-rays for the NT-20 sample (4.0 mg/mL, PBS). The SO increment has been monitored by recording the PL of the SO optical probe SOSG under simultaneous CW laser excitation at 473 nm (inset). (e) NT-to-PS energy transfer yield (ϕ_ET_^nr^, dots), relative scintillation yield () of the PS, and SO relative sensitization ability  after 600 s of exposure to soft X-rays for the multicomponent nanoscintillator series, as a function of the NT-to-PS intermolecular distance. Error bars are put as the mean standard deviation calculated on a *N* = 3 measurement replica.

The successful functionalization of the NT surfaces with the heterobifunctional chains and fluorescent PS molecules has been confirmed by means of vibrational and optical spectroscopy experiments. Figure 2c reports the infrared spectra of the bare NTs and the NT-*x* sample series. In all spectra, we can observe the main chrysotile vibrational peaks located at around 3700 cm^–1^ (MgOH stretching) and in the region around 1000 cm^–1^ (Si–O–Mg, Si–O–Si, and Si–O stretching).^37^ Furthermore, the spectra of the samples from NT-17 to NT-37 show clearly the peaks related to the Rhodamine Red C_2_ maleimide or to the rhodamine B functionalities (the C=C stretching vibrations at 1628 and 1542 cm^–1^, the N–C bending at 1291 cm^–1^, and the C–H stretching in the region around 3000 cm^–1^).^38,39^ The average number of PS molecules ⟨n⟩ coupled to each NT has been evaluated by means of optical absorption measurements (Table S1, Figure S1). Under UV excitation at 250 nm, all the functionalized NTs show a multiband PL spectrum (Figure 2d) where a residual NT emission at 430 nm can be observed, even very weak in some cases due the occurrence of ET_nr_. The PS fluorescence around 600 nm from the Rhodamine Red C_2_ maleimide and at 580 nm from the rhodamine B can be clearly distinguished in samples NT-17–NT-37 and samples NT-5* and NT-46*, respectively. No change in the emission properties is observed after keeping NT-*x* in a phosphate buffered saline (PBS) dispersion for up to 6 months, thus demonstrating the excellent stability of the synthesized materials. The residual NT PL intensity at 430 nm increases as a function of the NT-to-PS distance, thus suggesting the progressive reduction of ϕ_ET_^nr^ by separating the NT from the PS. ϕ_ET_^nr^ has been quantitatively evaluated by means of time-resolved PL experiments. Figure 2e shows the PL intensity decay in time of the samples monitored at 430 nm as a function of the NT-to-PS intermolecular distance. As expected, the emission decay accelerates by shortening the intermolecular distance that increases the ET_nr_ rate, which becomes competitive with the spontaneous recombination of the NT excited state.^33^ Both the bare NTs and the NT-*x* sample series show emission intensity decays with a multi-exponential behavior. The characteristic lifetime is calculated as the average emission lifetime ⟨τ_*X*_⟩ (Table S1). The ϕ_ET_^nr^ value is then calculated as , where ⟨τ_NT_⟩ is the average lifetime of the bare NT emission. ϕ_ET_^nr^ is reduced from 70% down to 10% by increasing the NT-to-PS nominal intermolecular distance from 5 to 46 Å (vide infra, Figure 3e).

Figure 3a shows the RL spectra of the NT and NT-*x* sample series powders (16 mg) recorded under steady-state excitation by soft X-rays (Experimental Methods). Similar to the PL spectra, also in this case, we can observe clearly the typical PS luminescence with a residual blue luminescence from NTs. The relative scintillation yield of the PS dyes  is taken as the RL intensity integrated in the PS emission spectral range (*I*_RL_^PS^). Notably, the PS scintillation luminescence is slightly red-shifted with respect to the PL spectrum in dispersion due to the enhanced self-absorption of dyes and the possible formation of aggregates in the powder form. Figure 3b shows the corresponding scintillation pulses recorded at the dye emission wavelength by exposing the powders to a pulsed X-ray source (Experimental Methods, Table S2). This experiment has been performed to have a hint on the luminescence properties of the materials under exposure to ionizing radiation. These measurements have been performed on powders because the pulsed X-ray source irradiance is too weak to record reliable signals from the diluted aqueous suspensions employed to generate the SO. For the samples from NT-5* to NT-24, the scintillation pulse lifetime is around 3 ns, with no significant differences. The observed values are slightly higher with respect to the corresponding PS PL decay time (Figure S3), in agreement with the possible self-absorption delay effect of the dye on the apparent emission lifetime. On the other side, the formation of low-energy J-aggregates is most probably responsible for the longer-emission component in the scintillation of samples NT-30, NT-37, and NT-46* and for the fast quenching observed in samples NT-37 and NT-46*.^36^ The more marked presence of aggregates in these samples agrees with the presence of long and more flexible surface ligands, which allows the connected dyes to interact more freely with respect to the NT functionalized with shorter ligands, which keep the dyes far enough to limit detrimental intermolecular interactions, especially in the powder form.

On the other hand, considering that the NT will be used in diluted aqueous dispersion, for a quantitative and reliable comparison between the different samples, we have measured the recombination kinetics of PS PL in the aqueous dispersion where they will be used to sensitize the SO production. Figure 3c reports the NT-*x* PL intensity decay with time recorded at 620 nm under pulsed laser excitation at 250 nm (Experimental Methods). In all cases, we observe an average decay time shorter than the one observed for the single chromophore in diluted solution (Figure S4), again most probably due to the presence of quenching J-aggregates on the NT surface, but there is no evident coherent trend. In some cases, the emission intensity decays as a single exponential function in a time shorter than the spontaneous one (1.97 ns for Rhodamine Red C_2_ maleimide and 2.71 ns for rhodamine B, Figure S4), while in some cases, we observe also a multi-exponential decay behavior (N-17, NT-19, and NT-37). Nevertheless, independently from its origin, the observed partial emission quenching suggests that, upon functionalization, the PL yield ϕ_pl_^PS^ (Experimental Methods, Table S3) of the PS is reduced. This means that the recombination properties of the PS singlet excited state are modified upon binding to NTs, including the intersystem crossing (ISC) rate that populates the triplet state from which the SO sensitization occurs by energy transfer to the ground-state molecular oxygen in solution (Figure 1b)^17^ and the triplet state lifetime that also affects the transfer to molecular oxygen. Therefore, also, the SO generation efficiency can be affected. This effect has been taken into account (vide infra) in order to have a reliable relative comparison of the samples ϕ_SO_.

ϕ_SO_ has been directly observed by the measurement of the relative SO production efficiency under soft X-ray exposure. Figure 3c reports the evolution of the SO concentration in PBS dispersion of NT-20, as an example, which has been monitored in situ by using the SO Sensor Green (SOSG, Experimental Methods) as an optical probe.^20^ The SOSG PL intensity is proportional to the concentration of SO;^40^ thus, upon its selective excitation, we can compare the evolution of the SO concentration as a function of time (inset of Figures 3c and S5). Specifically, ϕ_SO_ is defined here as the relative increment of the SO concentration, and it is calculated as . All the samples in the series have been monitored under steady-state X-rays exposure up to 600 s which corresponds to a delivered dose of approximately 260 Gy, in glass vials. All the samples show a SO sensitization ability (Figure S4). In order to have a reliable relative comparison, the SO sensitization efficacy is finally calculated as a relative normalized SO sensitization ability , thus taking into account the perturbation of the PS observed upon binding that is assumed to modify its ϕ_pl_^PS^ and thus indirectly the SO ability.

The comparative analysis among the observed ϕ_ET_^nr^, , and  as a function of surface ligand length is reported in Figure 3e. It is worth noting that the absorption of the PS molecules in the investigated dispersions is very low (Figure S8), and the NT emission efficiency is very weak (≪5%) so that we can exclude a priori a relevant photoexcitation of the conjugated PS by direct absorption of the NT scintillation light (i.e., radiative energy transfer). As discussed above, the ϕ_ET_^nr^ value decreases by about 1 order of magnitude by moving progressively far away the PS molecules from the scintillating NT, until a ϕ_ET_^nr^ = 10% is observed in the NT-46* sample in agreement with the distance-dependent behavior of the non-radiative ET_nr_ rate. On the other side, both  and  show a substantially constant behavior completely uncorrelated to ϕ_ET_^nr^, thus suggesting that the scintillation light output and the efficiency of the SO sensitizer are independent on the system architecture. Even in the best configuration with ϕ_ET_^nr^ = 70%, no enhancement is observed in the SO production. Similar results are observed by using a different optical probe for the SO formation (Figures S6 and S7). These findings demonstrate therefore the negligible role of ET_nr_ between the nanoscintillator NTs and the PS moiety in activating the SO sensitization ability of multicomponent materials for PDT-enhanced radiotherapy. Moreover, these results confirm experimentally for the first time the output of radiation/matter interaction simulations in nanoscintillators recently proposed.^41^ According to the dimension of our nanoscintillators, only a minor fraction of the energy deposited upon interaction of the X-rays with the high-Z elements is stored in the particle itself, while most of the energy is spread around the particle by generating a swarm of secondary charges that can diffuse for distance up to hundreds of nanometers. Thus, even in the best case where ϕ_ET_^nr^ equals unity, the effective boosting of the PDT activity due to the ET_nr_ channel can be only negligible, while the major role in the global energy partitioning process is played by the direct recombination of free charges on the PSs, which is locally sensitized by the presence of the dense nanoscintillator.

## 3 Conclusions

In conclusion, we successfully realized a series of multicomponent nanoscintillators as a model system for PDT-enhanced radiotherapy coadjutants. Their architecture has been finely tailored in order to control the efficiency of the non-radiative energy transfer process between the building blocks of the multicomponent system, namely, the scintillating dense nanoparticle, responsible for the localized interaction with the ionizing radiation, and the attached ROS-sensitizing PS species that enable the PDT. The obtained results demonstrate that the non-radiative energy transfer plays a marginal role in the global energy partitioning process responsible for the evident synergistic effect of radiotherapy and deep-tissue X-ray-activated PDT usually observed during cancer treatment using these materials. This finding has important consequences, by pointing out some new guidelines pivotal for the design and realization of optimized multicomponent radiotherapy coadjutants. First, the match between the electronic transitions of the scintillator and the PS is no more strictly required since the PS is mainly activated by direct recombination of the free charges produced during the primary and secondary interaction events in the scintillation process. This strongly relaxes the constraints on the type of PS that can be used. Second, the close packing of scintillators and PSs is no more required to maximize the energy transfer rate, thus again significantly relaxing the constraints on the system architectures and avoiding the problems originating from the need for specific control of intermolecular interactions between close-packed species. Third, considering that heaviest elements such as lead could represent a critical issue for their poor biocompatibility, the obtained results indicate that larger but still biocompatible nanoparticles are required to maximize the local radiosensitization effect in tumors. For example, hafnia and/or zirconia nanoparticles^7,42,43^ with size up to 100–200 nm can be envisaged. According to the obtained results, the best arrangement for the PS moiety could be, for example, a shell wrapped around the dense nanoparticle with thickness up to 100 nm, in order to harvest the most of the diffusing charge energy. This design will result in a bigger multicomponent system with still good cellular uptake and delivery in the body^27,44−47^ and a simultaneous good interaction with the ionizing radiations and optimized energy harvesting and partitioning that will potentially lead to a breakthrough increment of the radiotherapy effect even at low doses.

## 4 Experimental Section

### 4.1 Synthesis of Stoichiometric Chrysotile Nanotubes

Chrysotile NTs were synthesized according to a previously used synthetic method.^20^ A hydrothermal reactor with a 100 cm^3^ polypropylene vessel was used to carry out the hydrothermal reaction of 1522 mg of Na_2_SiO_3_ and 764 mg of MgCl_2_ in an aqueous solution of NaOH (220 mL 0.4 M) at 250 °C with a run duration of 16 h. The precipitate removed from the solution was repeatedly washed with deionized water before being dried for 3 h at 110 °C.

### 4.2 Functionalization of Chrysotile Nanotubes with Chains of Different Lengths

For the preparation of each sample, 100 mg of NT powder was suspended in 25 mL of PBS and 30 mg of 16-mercaptohexadecanoic acid suspended in 25 mL of PBS or 40 mg of 11-mercaptoudecanoic acid suspended in 20 mL of PBS or 810 μL of 8 mercaptooctanoic acid, or 600 μL of 3 mercaptopropionic acid, or 120 mg of l-cysteine suspended in 25 mL of PBS , or 620 μL of thioglycolic acid were added slowly under stirring for 10 min. Samples were centrifuged for 5 min at 6500 rpm. The precipitate removed from the solution was repeatedly washed with deionized water before being dried for 3 h at 50 °C.

### 4.3 Functionalization of NTs + Variable Length Chain with Invitrogen Rhodamine Red C_2_ Maleimide

40 mg of NTs functionalized with chains of different lengths was dispersed in 5 mL of tris(hydroxymethyl)aminomethane (TRIS), and 4 mL of Invitrogen Rhodamine Red C_2_ maleimide (1.4 mg in 70 mL of TRIS) was added in the solution. Maleimide is a thiol-reactive probe and reacts with thiol groups in a typical thiol–maleimide “click” chemistry reaction to give thioether-coupled products. Samples were centrifuged for 5 min at 6500 rpm. The precipitate removed from the solution was repeatedly washed with deionized water and PBS before being dried for 3 h at 50 °C.

### 4.4 Functionalization of Chrysotile Nanotubes with Rhodamine B

60 mg of NTs was dispersed in 15 mL of PBS, and 2 mL of rhodamine B (3 × 10^–5^ M in PBS) was added in the solution. Samples were centrifuged for 5 min at 6500 rpm. The precipitate removed from the solution was repeatedly washed with deionized water and PBS before being dried for 3 h at 50 °C.

### 4.5 Functionalization of Chrysotile Nanotubes with Rhodamine B-PEG2k-COOH (Sigma-Aldrich)

60 mg of NTs was dispersed in 15 mL of PBS, and 2 mL of rhodamine B- PEG2k-COOH (2 mg/7 mL PBS) was added in the solution. Samples were centrifuged for 5 min at 6500 rpm. The precipitate removed from the solution was repeatedly washed with deionized water and PBS before being dried for 3 h at 50 °C.

### 4.6 Diffraction Experiment (XRD)

Powder X-ray diffraction patterns were acquired in Bragg–Brentano geometry with Cu Kα radiation (analytical X’Pert Pro powder diffractometer).

### 4.7 Transmission Electron Microscopy

Transmission electron microscopy (TEM) observations have been performed with a JEOL JEM1220. TEM samples were prepared by dispersing a few milligrams of the compounds in 2 mL of distilled water and dropping 3 μL of solution on carbon-coated copper grids.

### 4.8 Attenuated Total Reflection Fourier-Transform Infrared Spectroscopy

Attenuated total reflection Fourier-transform infrared spectroscopy spectra of dried samples were obtained on a Thermo Scientific Nicolet iS20 FTIR spectrometer.

### 4.9 Optical Studies

Absorption spectra were recorded using a Cary Lambda 900 spectrophotometer at normal incidence with Suprasil quartz cuvettes with a 0.1 cm optical path length. Steady-state PL and PL excitation spectra have been recorded using a xenon lamp as an excitation source, together with a double monochromator (Jobin-Yvon Gemini 180 with a 1200 grooves/mm grating), and recorded through a nitrogen-cooled charge-coupled device (CCD) detector coupled to a monochromator (Jobin-Yvon Micro HR). Under cw laser excitation, signals have been recorded using a nitrogen-cooled CCD coupled with a double monochromator, Triax- 190 (HORIBA Jobin-Yvon), with a spectral resolution of 0.5 nm. All spectra have been corrected for the setup optical response. Time-resolved PL spectra have been recorded using a pulsed light-emitting diode (LED) at 250 nm (3.65 eV, EP-LED 340 Edinburgh Instruments, a pulse width of 700 ps) or a pulsed laser at 405 nm (3.06 eV, EPL-405 Edinburgh Instruments, a pulse width of 150 ps) as a light source. Data were obtained with an Edinburgh Instruments FLS-980 spectrophotometer, with a 5 nm bandwidth and a time resolution of 0.1 ns.

### 4.10 Radioluminescence Experiments

RL measurements were performed by irradiating the samples at room temperature with a Philips 2274 (steady-state RL spectroscopy) or a Machlett OEG 50 (SO production monitoring experiment) X-ray tubes, both with a tungsten target, equipped with a beryllium window and operated at 20 kV and 20 mA. At this voltage, X-rays are generated by the bremsstrahlung mechanism superimposed onto the L and M transition lines of tungsten due to the impact of electrons generated through the thermionic effect and accelerated onto a tungsten target. No beam filtering has been applied. RL spectra have been recorded using a homemade apparatus featuring a liquid nitrogen-cooled CCD (Jobin-Yvon Symphony II) coupled to a monochromator (Jobin-Yvon Triax 180) with a 100 grooves/mm grating as the detection system. The spectra were corrected for the setup optical response. For RL experiments, the NT-*x* powder was used to fill small aluminum crucibles of 1 mm thickness to completely absorb the incident X-rays. Therefore, in all samples, we have the same amount of deposited energy. Therefore,  is directly given by the ratio of the integrated intensity of the RL spectra.

### 4.11 Scintillation Experiments

Scintillation decays under pulsing X-ray excitation were measured at room temperature using picosecond (ps) X-ray tube N5084 (Hamamatsu Photonics, Japan) at 40 kV. The X-ray tube was driven by the ps light pulse from a laser with a repetition rate of up to 1 MHz. The signal was detected by a hybrid ps photon detector and Fluorohub unit (Horiba Scientific, Japan). The setup instrumental response function full width at half-maximum was about 70 ps. The scintillation decay curves were detected using a high-pass filter for the range above 580 nm. The emission was monitored from the same sample’s surface where it was excited.

### 4.12 SO Relative Concentration Measurement

The optical probe SOSG has been purchased from Thermo Fisher and used as is. The SOSG powder has been diluted in a 1:10 solution of dimethyl sulfoxide and PBS, which has been used to disperse the NTs with a concentration of 4 mg/mL. The intensity of the SOSG fluorescence, which is directly proportional to the concentration of SO in the environment, has been monitored during the X-ray exposure under continuous-wavelength laser light excitation at 473 nm. The integrated SOSG PL is then proportional to the amount of SO produced upon irradiation. The SOSG emission intensity was integrated between 500 and 530 nm, in order to avoid inclusion of the emission of the PSs. The measured values have been corrected by the dye quantum yield, by the relative intrinsic efficiency of SO generation of the two rhodamines (Figure S2), and by the average number of dyes per NT..

## Supporting Information Available

Optical, scintillation, and SO sensitization experiments (PDF)
