# Supplementary material for: Energy Partitioning in Multicomponent Nanoscintillators for Enhanced Localized Radiotherapy
Source: ACS Appl Mater Interfaces. 2023 May 12;15(20):24693–700. doi: 10.1021/acsami.3c00853 (PMC10214376; doi:10.1021/acsami.3c00853)
Supplement: Supplementary file 1 — am3c00853_si_001.pdf [file am3c00853_si_001.pdf]

Supporting Information for

Energy partitioning in multicomponent nanoscintillators for enhanced localized radiotherapy

Valeria Secchi[1,2], Francesca Cova[1], Irene Villa[3†], Vladimir Babin[3], Martin Nikl[3], Marcello Campione[4,2], Angelo Monguzzi[1,2]*

[1] Dipartimento di Scienza dei Materiali, Università degli Studi Milano-Bicocca, 20125 Milano, Italy

[2] NANOMIB, Center for Biomedical Nanomedicine, University of Milano-Bicocca, P.zza Ateneo Nuovo 1, 20126 Milan, Italy

[3] FZU – Institute of Physics of the Czech Academy of Sciences, Cukrovarnická 10/112, 162 00 Prague, Czech Republic

[4] Department of Earth and Environmental Sciences, Università degli Studi Milano-Bicocca, Piazza della Scienza 4, 20126 Milano, Italy

[†] current address: Dipartimento di Scienza dei Materiali, Università degli Studi Milano-Bicocca, 20125 Milano, Italy

Corresponding Author:
Angelo Monguzzi angelo.monguzzi@unimib.it

Table S1. Characteristic of the functionalized NT-x samples series investigate in this study. Intermolecular distances have been calculated by the Avogadro software.[1]

Sample	Ligands	Ligand Molecular Structure	Photosens. (PS)	NT-PS distance (Å)	NT PL lifetime @ 430 nm (ns)	Energy transfer efficiency	Dyes / NT
**NT**	-	-	-	0	6.1	---	
**NT-5***	-	-	Rhodamine B	5	1.9	0.69	652.1
**NT-17**	Thioglycolic acid	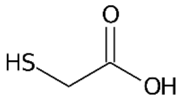	Rhodamine Red™ C2 Maleimide	17	2.6	0.57	705.4
**NT-19**	L-Cysteine	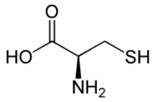	Rhodamine Red™ C2 Maleimide	19	2.8	0.54	252.5
**NT-20**	3-Mercaptopropionic acid	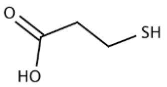	Rhodamine Red™ C2 Maleimide	20	3.8	0.41	457.6
**NT-24**	8-mercaptooctanoic acid	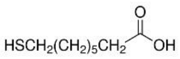	Rhodamine Red™ C2 Maleimide	24	3.7	0.38	157.6
**NT-30**	11-mercaptoundecanoic acid	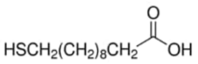	Rhodamine Red™ C2 Maleimide	30	3.6	0.36	564.0
**NT-37**	16-mercaptohexadecanoic acid	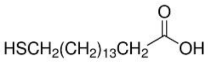	Rhodamine Red™ C2 Maleimide	37	4.7	0.23	402.6
**NT-46***	Polyethylene glicol (PEG)	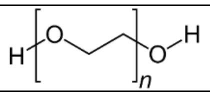	Rhodamine B	46	5.3	0.10	502.6

Supplementary Data

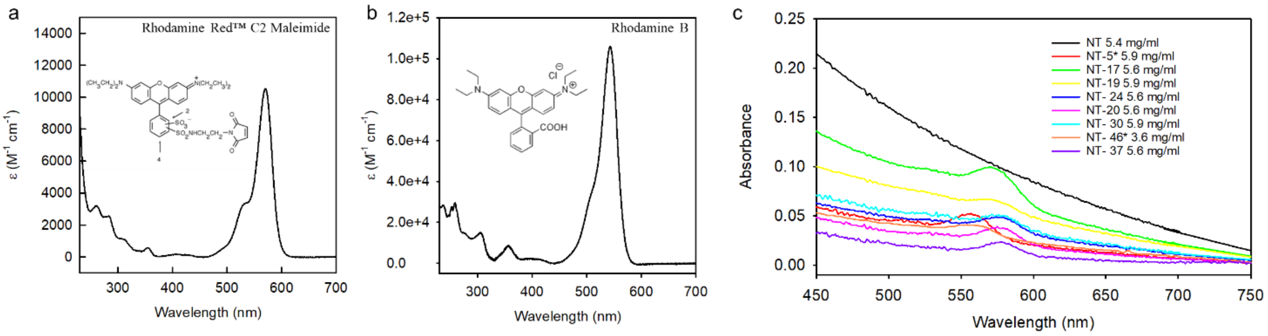

Figure S1. Molar extinction coefficient for Rhodamine Red™ C2 Maleimide (a) and Rhodamine B (b) dyes. Panel c show the absorption spectra recorded at normal incidence of the functionalized NT series in H_2O dispersion (1 cm optical path).

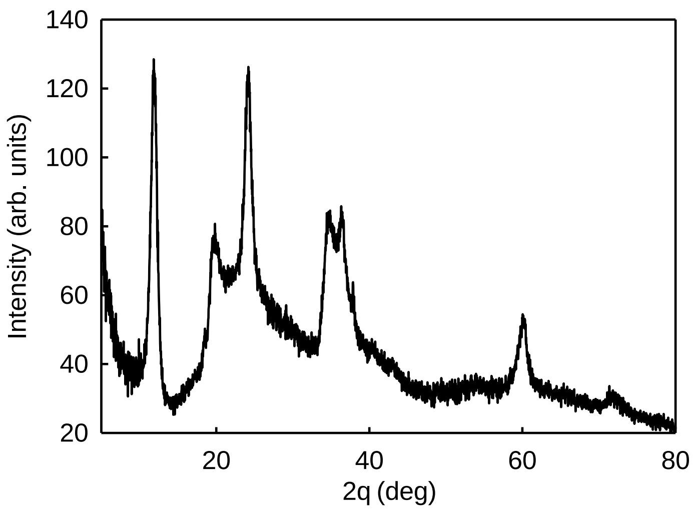

Figure S2. PXRD patterns of a chrysotile NT, in agreement with previous results.[2]

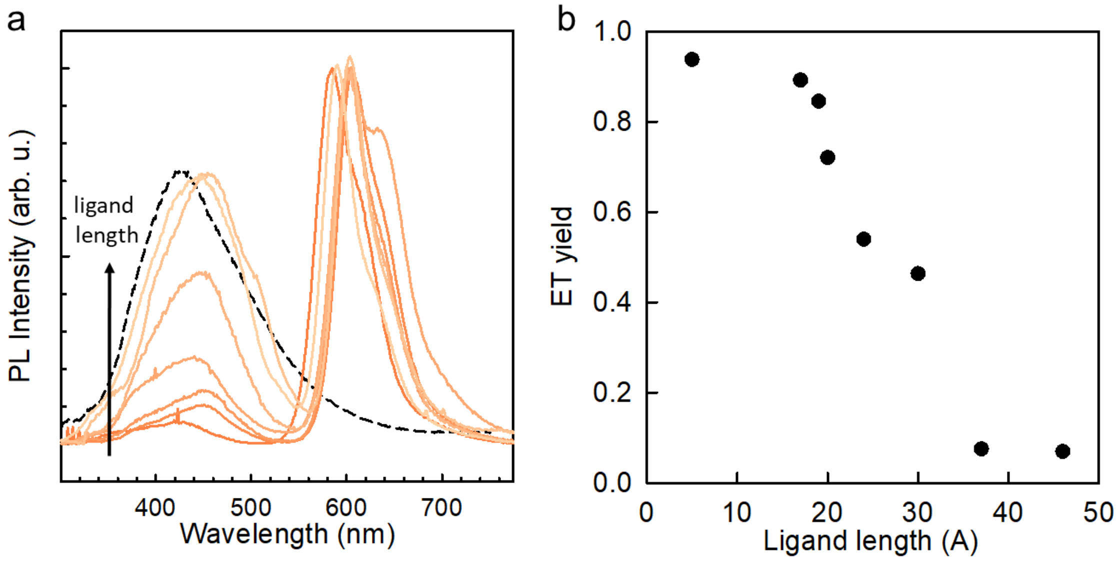

Figure S3. a. PL spectra of multicomponent nanoscintillators as a function of the NT-to-PS intermolecular distance under UV excitation at 250 nm. b. NT-to-PS non radiative energy transfer (ET_{nr}) yield calculated from the steady state PL measurements reported in the Fig.2d. The ET yield has been calculated as $1 - I(x)/I_0$ where I_0 is the NT emission intensity in absence of PS and *I(x)* is their emission intensity in presence of PS as function of their intermolecular distance (Table S1). The PL intensity has been calculated as the integer of the emission spectrum. It should be noted that the investigated NT solutions are highly scattering samples, so steady state PL measurements are not completely reliable to evaluate the non-radiative energy transfer yield, because of potential parasitic effects such as the direct absorption of scattered photon trapped in the solution by photosensitizers. Therefore, these calculations can result an overestimation of the non-radiative energy transfer yield as demonstrated by the more accurate time resolved PL experiments reported in Fig. 2e.

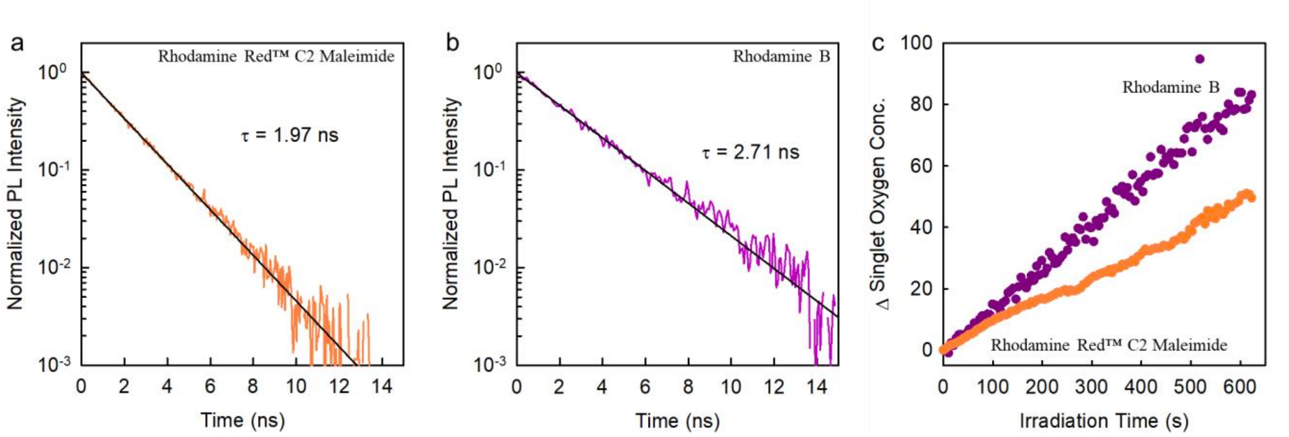

Figure S4. Properties of Rhodamine Red™ C2 Maleimide and Rhodamine B dyes. a, b. Photoluminescence (PL) intensity decay under pulsed excitation at 250 nm in PBS solution. c. Relative increment of singlet oxygen concentration as a function of the irradiation time under soft x-rays in PBS scattering solution with PMMA particles.

Table S2. Multi-exponential fit parameters for the scintillation pulses recorded at 600 nm and showed in Fig. 3b. Time values are reported in nanoseconds.

	A_1	τ_1	A_2	τ_2	$< \tau >$
**NT-5***	0.58	2.06	0.42	23.09	3.40
**NT-17**	0.68	2.45	0.32	17.62	3.40
**NT-19**	1.00	3.24	-	-	3.70
**NT-20**	0.61	1.30	0.39	45.71	2.51
**NT-24**	0.56	1.77	0.44	19.60	2.90
**NT-30**	0.67	15.2	0.33	-	-
**NT-37**	0.51	0.51	0.49	16.7	0.90
**NT-46***	0.70	0.35	0.30	18.9	0.52

Table S3. Multi-exponential fit parameters for the photoluminescence intensity decays recorded at 600 nm and showed in Fig. 3c. Time values are reported in nanoseconds.

	A_1	τ_1	A_2	τ_2	A_3	τ_3	$< \tau >$
**NT-5***	1.00	1.54	-	-	-	-	-
**NT-17**	0.50	0.73	0.33	1.82	0.17	2.31	1.38
**NT-19**	0.55	0.81	0.45	2.13	-	-	1.34
**NT-20**	1.00	1.84	-	-	-	-	-
**NT-24**	1.00	1.14	-	-	-	-	-
**NT-30**	1.00	1.74	-	-	-	-	
**NT-37**	0.94	1.55	0.06	4.7	-	-	1.74
**NT-46***	1.00	1.75	-	-	-		

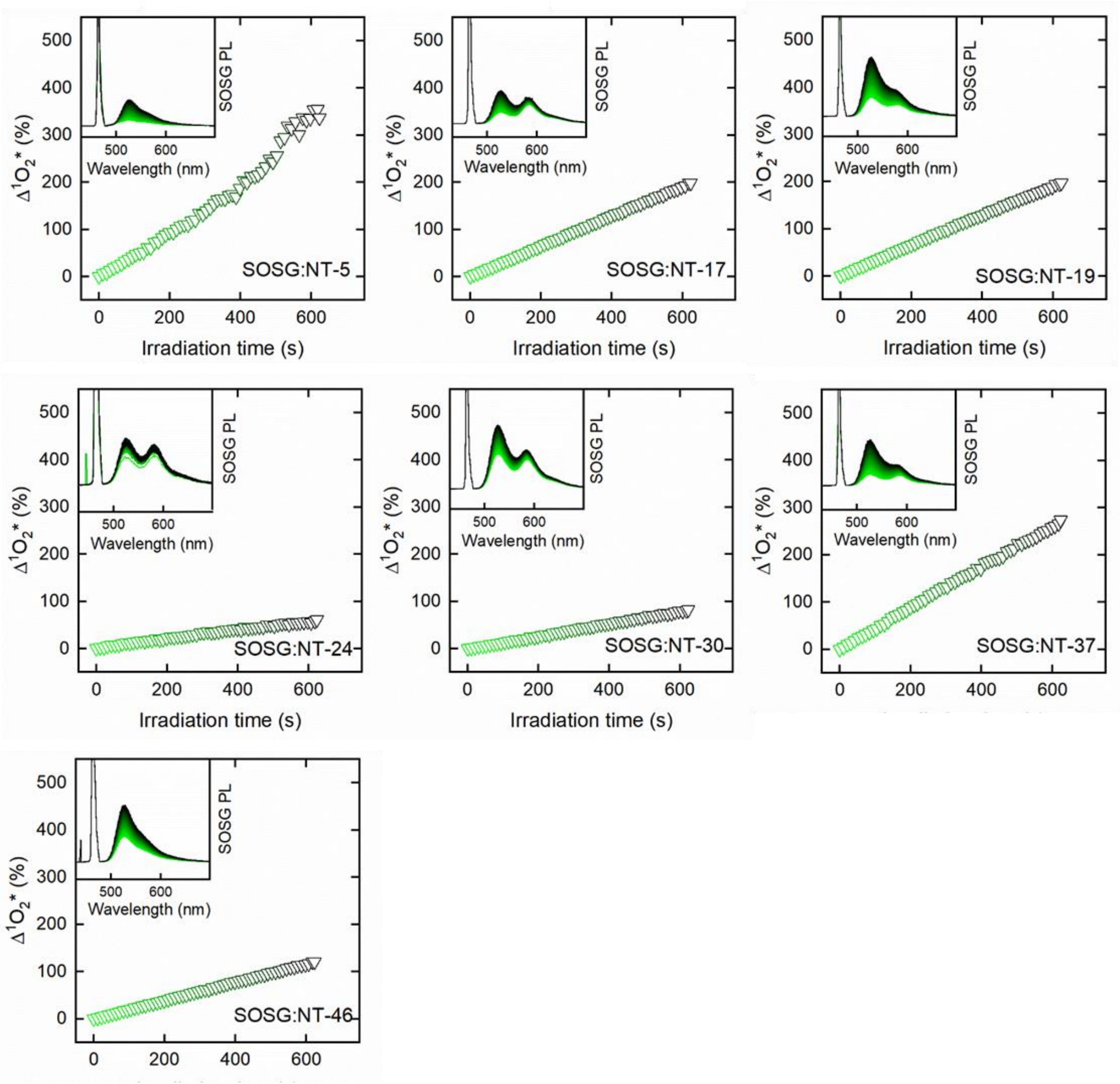

Figure S5. Relative increment of singlet oxygen concentration as a function of the irradiation time under soft x-rays for the NT-x sample series in PBS dispersion (4.0 mg/ml). The insets report the PL of the singlet oxygen optical probe SOSG as a function of the irradiation time with soft x-rays and under CW laser excitation at 473 nm. The SOSG emission intensity reported in the main plots has been integrated between 500 and 530 nm, in order to avoid to include the emission of the photosensitizers at 580 nm (NT-5* and NT-46*) and 610 nm (NT-17, NT-19, NT-24, NT-30, NT-37).

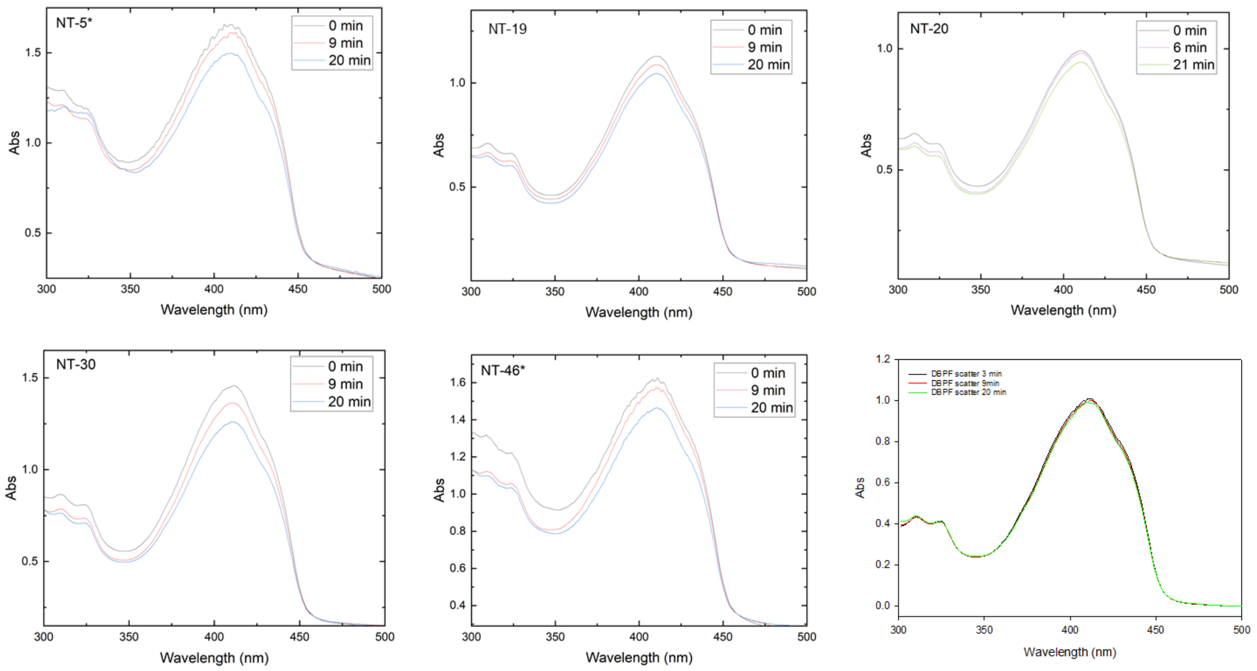

Figure S6. Relative increment of singlet oxygen concentration as a function of the irradiation time under soft x-rays for the NT-x sample series in H_20/EtOH (50/50) dispersion (1.3 mg/ml). As optical probe, we monitor the absorption of the DBPF at 412 nm, which reduces following the increment of the singlet oxygen concentration.[1] The NT-x dispersion have been prepared according the literature indication, but a strong fraction of EtOH is required because of the poor solubility of DBPF in water. NT-17, NT-24 and NT-37 samples are not reported here because of the appearance of critical stability problems in the new solution with EtOH. So the measurements upon irradiation are not reliable. Last panel show the response to irradiation of the optical probe alone in solution with inert PMMA microparticles taken as control.

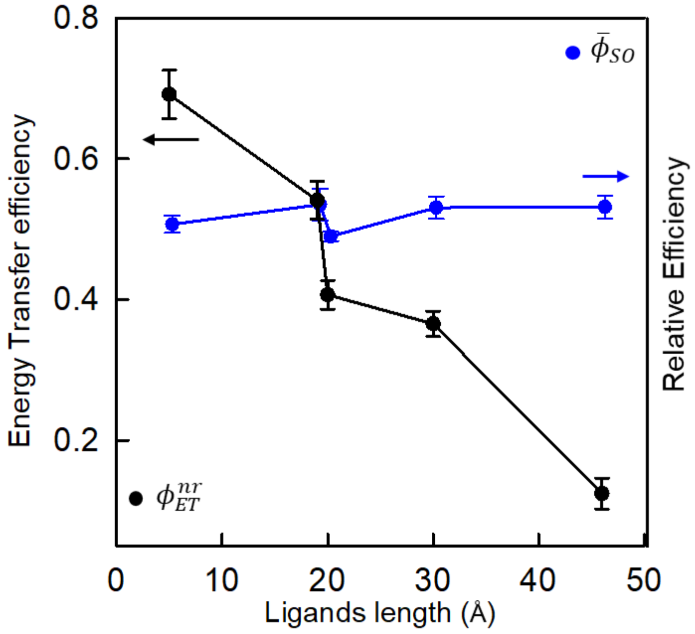

Figure S7. SO relative sensitization ability $\bar{\phi}_{SO}$ after 600 second of exposure to soft x-rays of delivered dose for the multicomponent nanoscintillators series, as a function of the NT-to-PS intermolecular distance. The $\bar{\phi}_{SO}$ has been calculated considering the reduction in the DBPF absorbance at 412 nm showed in Fig. S6 as a function of the irradiation time, normalized by the average number of molecule per NT (<*n*>) and by the dyes PL quantum yield, as described in the main text.

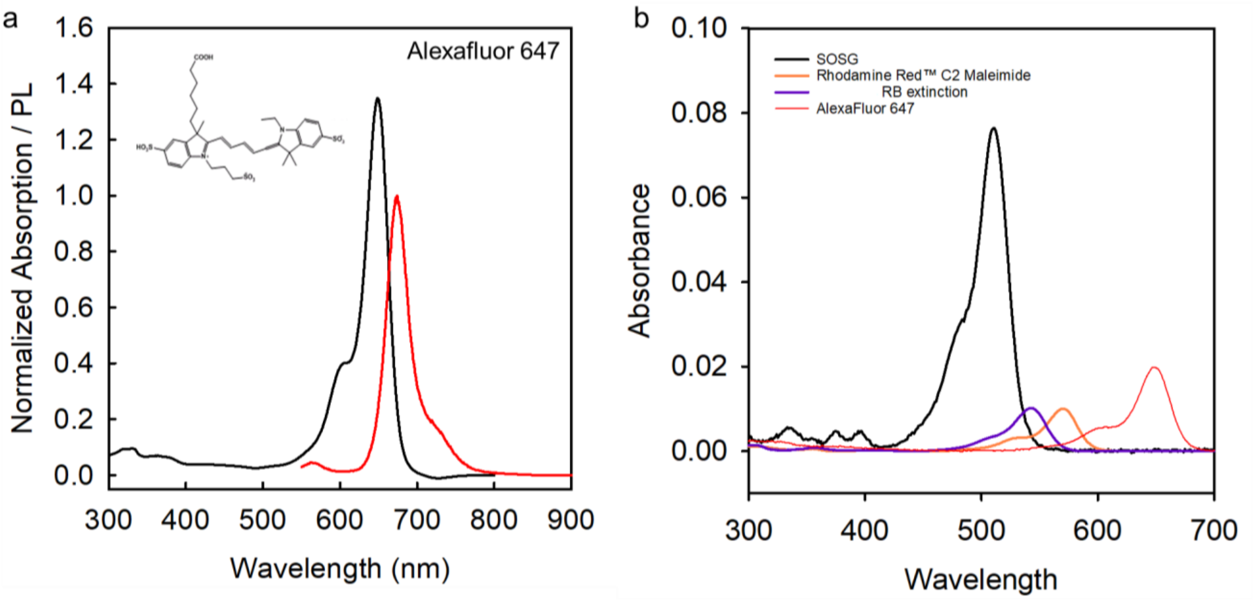

Figure S8. a. Absorption and PL spectra of the Alexafluor 647 dye employed as non-resonant PS system. b. Absorption spectra of the PS dyes employed in this work compared to the absorption spectrum of the SO optical probe SOSG, measured at the concentration employed in the experiments (optical path 1 mm). The dyes absorption at the 473 nm laser excitation is negligible with respect to the SOSG one, so direct optical excitation of the PS is always negligible.

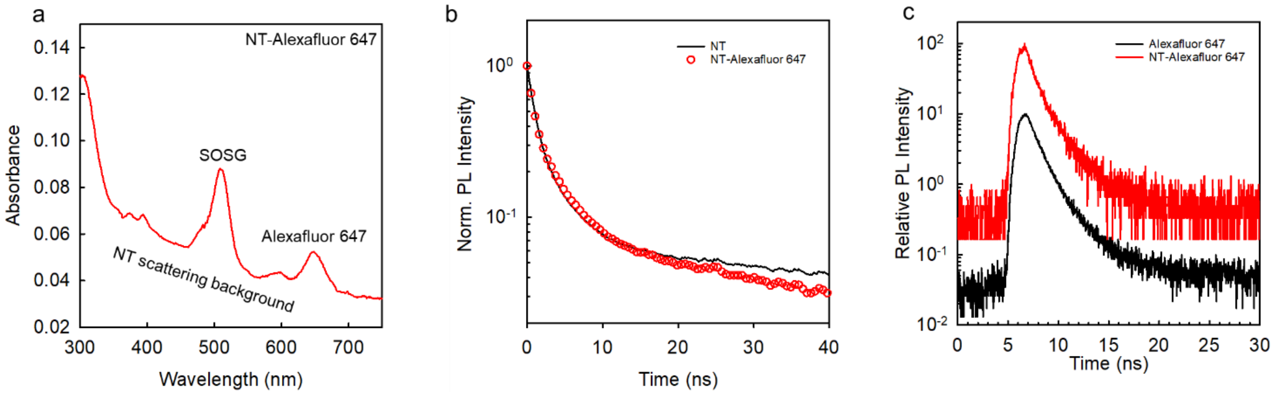

Figure S9. a. Absorption spectra recorded at normal incidence of the NT-Alexafluor 647 sample in dispersion with SOSG for singlet oxygen generation and monitoring (optical path 1 mm). The average number of dye per NT is <*n*> = 3320 b. PL intensity decay in time recorded at 430 nm under pulsed excitation at 250 nm of NTs and NT-Alexafluor 647 sample. No changes are observed, suggesting the absence of non-radiative energy transfer. c. PL intensity decay in time recorded at 700 nm under pulsed excitation at 250 nm of NTs and NT-Alexafluor 647 sample. No changes are observed in the dye emission lifetime upon binding to the NT surfaces, suggesting the absence of quenching processes that can affect the dye ability to sensitize the singlet oxygen.

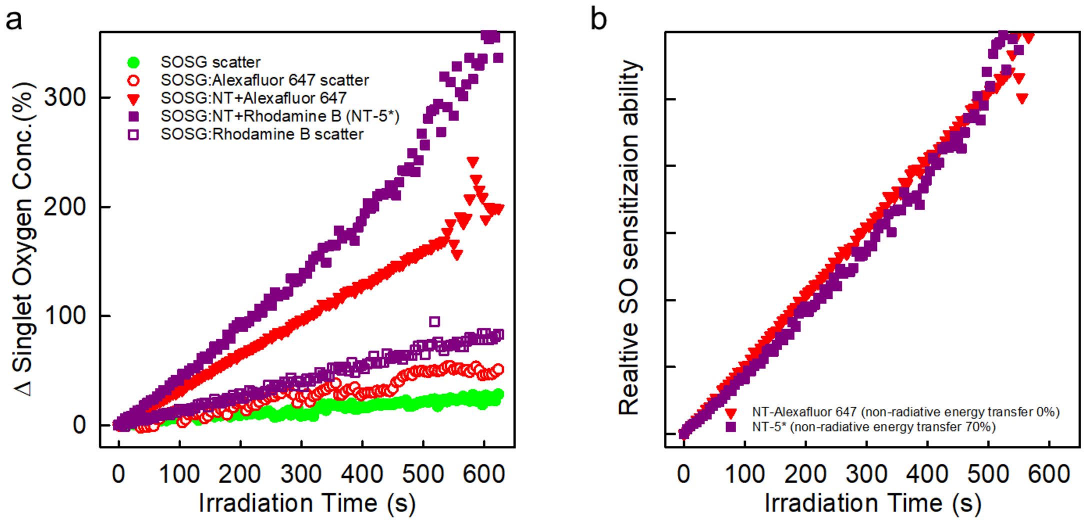

Figure S10. Relative increment of singlet oxygen (SO) concentration as a function of the irradiation time under soft x-rays for the SOSG, Rhodamine B and Alexafluor 647 in dispersion with PMMA particles for light scattering, and NT-5* and NT-Alexafluor 647 samples (6.0 mg/ml, PBS). The dye concentration is the same that the one attached on NTs. b. Relative SO oxygen sensitization ability calculated as described in the main text and corrected by the dyes SO generation ability reported in panel a. Notably, despite the absence of the energetic resonance between the NT emission and the Alexafluor 647, the SO is generated with that same efficiency upon irradiation.

Supplementary References

1. Avogadro: an open-source molecular builder and visualization tool. Version 1.20. http://avogadro.cc/ **2016**

2. Entradas, T.; Waldron, S.; Volk, M. Journal of Photochemistry and Photobiology B: Biology, The detection sensitivity of commonly used singlet oxygen probes in aqueous environments. **2020,** *204*, 111787.
